# Particulate and gaseous air pollutants exceed WHO guideline values and have the potential to damage human health in Faisalabad, Metropolitan, Pakistan

**DOI:** 10.1007/s10661-024-12763-3

**Published:** 2024-06-25

**Authors:** Nukshab Zeeshan, Ghulam Murtaza, Hamaad Raza Ahmad, Abdul Nasir Awan, Muhammad Shahbaz, Peter Freer-Smith

**Affiliations:** 1 Institute of Soil and Environmental Sciences, University of Agriculture, Faisalabad, 38040 Pakistan; 2 Department of Structures and Environmental Engineering, University of Agriculture, Faisalabad, 38040 Pakistan; 3 Department of Botany, University of Agriculture, Faisalabad, 38040 Pakistan; 4 Department of Plant Sciences, University of California, One Shields Avenue, Davis, CA 95616 USA

**Keywords:** Particulates, CO, NO_2_, SO_2_, Heavy metals, Human health, Air quality index

## Abstract

First-ever measurements of particulate matter (PM_2.5_, PM_10_, and TSP) along with gaseous pollutants (CO, NO_2_, and SO_2_) were performed from June 2019 to April 2020 in Faisalabad, Metropolitan, Pakistan, to assess their seasonal variations; Summer 2019, Autumn 2019, Winter 2019–2020, and Spring 2020. Pollutant measurements were carried out at 30 locations with a 3-km grid distance from the Sitara Chemical Industry in District Faisalabad to Bhianwala, Sargodha Road, Tehsil Lalian, District Chiniot. ArcGIS 10.8 was used to interpolate pollutant concentrations using the inverse distance weightage method. PM_2.5_, PM_10_, and TSP concentrations were highest in summer, and lowest in autumn or winter. CO, NO_2_, and SO_2_ concentrations were highest in summer or spring and lowest in winter. Seasonal average NO_2_ and SO_2_ concentrations exceeded WHO annual air quality guide values. For all 4 seasons, some sites had better air quality than others. Even in these cleaner sites air quality index (AQI) was *unhealthy for sensitive groups* and the less good sites showed *Very critical* AQI (> 500). Dust-bound carbon and sulfur contents were higher in spring (64 mg g^−1^) and summer (1.17 mg g^−1^) and lower in autumn (55 mg g^−1^) and winter (1.08 mg g^−1^). Venous blood analysis of 20 individuals showed cadmium and lead concentrations higher than WHO permissible limits. Those individuals exposed to direct roadside pollution for longer periods because of their occupation tended to show higher Pb and Cd blood concentrations. It is concluded that air quality along the roadside is extremely poor and potentially damaging to the health of exposed workers.

**Graphical abstract:**

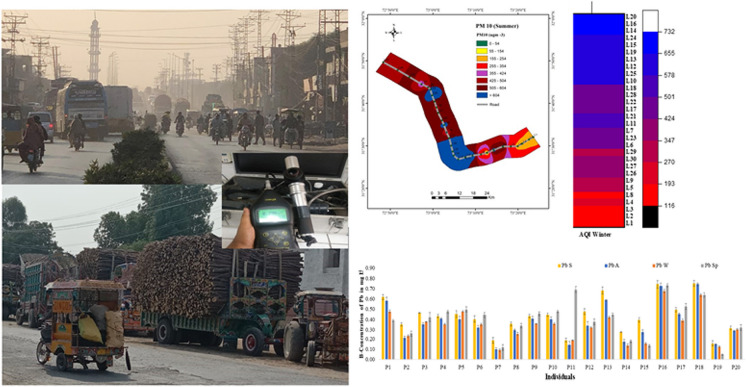

**Supplementary Information:**

The online version contains supplementary material available at 10.1007/s10661-024-12763-3.

## Introduction

Air pollutants are substances that are present in the air at concentrations that can harm humans, plants, and the environment. Some pollutants have both natural and anthropogenic sources. Anthropogenic emissions arise from transportation, industrial activities, agriculture, and energy production (Capes et al., 2009). The most common air pollutants are particulate matter (PM), nitrogen and sulfur oxides (NOx, SOx), carbon mono- and dioxide (CO, CO_2_), ozone (O_3_), and volatile organic compounds (VOCs). According to the World Health Organization (WHO, 2021), 9 out of 10 people breathe air that contains high concentrations of pollutants, and exposure results in an estimated 7 million premature deaths every year. The Global Burden of Disease Study found that outdoor air pollution is responsible for 4.2 million deaths every year, with fine particulate matter (PM_2.5_) contributing the most (Cohen et al., 2017). PM are categorized based on particle diameter in micrometers (µm); PM_2.5_, PM_4_, and PM_10_, collectively referred to as total suspended particulates (TSP). Carbon monoxide (CO) is a silent killer if inhaled and the United States Environmental Protection Agency (US-EPA) sets an 8-h average value at 9 ppm for this pollutant (US-EPA, 2015). In the absence of enough oxygen, incomplete combustion of fuel in vehicles, boilers, or incinerator fuels yields CO (Rossner et al., 2017), causing higher CO concentrations in their immediate vicinities. CO interrupts oxygen delivery to the organs and tissues of the human body. During the COVID-19 pandemic, air pollutant concentration was reduced significantly due to reduced transportation and industrial activities, indicating that there is a direct relationship between pollutant emissions and fossil fuels combustion (Sari & Kuncoro, 2021). NOx and SO_2_ are also produced by the combustion of fossil fuels and are thus emitted by vehicle exhausts, and from industrial chimneys (Javed et al., 2014; Kumar et al., 2018; Richter et al., 2005). Emission standards are available at US-EPA website for all pollutants (US-EPA, 2015). High concentrations of NOx and SO_2_ pollutants impact human health (Nukshab et al., 2020) causing lung irritation, acute respiratory illness, cardiovascular diseases, respiratory illness, bronchitis, emphysema, and reduction of gestational age (Anderson et al., 2018; Behrentz et al., 2004; Cakmak et al., 2014). Their emissions become higher in congested areas where more vehicles pass per unit of time (Liu et al., 2018; Men et al., 2018).

When ambient air pollution is monitored at a specific site over a predetermined period (such as 1 or 24 h), the results reported internationally are known as air quality index (AQI); with a grading scale ranging from good to very critical categories. The primary goals of the AQI are to enforce obligatory regulatory measures and to inform and warn the public about the risks associated with daily exposure to pollution levels (Gurjar et al., 2008). The air quality may be monitored and managed using the GIS-based air pollution mapping (interpolation), which has been shown to be an effective visualization tool that can help locate pollution hotspots and potential sources. The carbon content of particulates is not only directly injurious to health, but also binds other chemicals and transfers them to the human body. It has been shown that the sulfur content of dust produced from vehicular exhausts is high (Hernández-Terrones et al., 2020) and sulfur is dangerous for the skin and also causes irritation in the eyes (Bandowe et al., 2019). Almost 50% of secondary aerosol mass is made up of carbon and contains cadmium (Cd) and lead (Pb) which are dangerous to exposed organisms, i.e., humans (Liu et al., 2018; Men et al., 2018). Cd enters the body through respiration and becomes part of human blood. It is reported that Cd is taken up by the liver from the blood and is deposited and accumulates in the kidney. The heart, spleen, lungs, and bones are also accumulators of Cd (Ma et al., 2021). The half-life of Cd is reported to be about 20 years (Nordberg & Nordberg, 2022). It causes oxidative stress and due to this, tissues are damaged (de Bont et al., 2022). Pb is toxic to almost every organ of the body. Exposed adults and children are vulnerable to Pb contamination and exposure of urban populations has been shown to effect maternal foliate status and intergenerational risk of childhood obesity (Wang et al., 2019) It directly binds to the nervous system of adults and reduces the cognitive power of an individual (Rubin et al., 2008).

According to the State of Global Air 2020 report, Pakistan’s PM_2.5_ exposures are among the highest in the world, with an annual average concentration of 66 µg m^−3^, which exceeds the WHO guideline values (Annual average of 5 µg m^−3^ with 24-h limit being 15 µg m^−3^) by a substantial margin. The report estimates that air pollution causes around 135,000 premature deaths in Pakistan each year; placing Pakistan globally as the country with the 5th highest number of pollution-related deaths (https://www.stateofglobalair.org/resources/countryprofiles?countrychoice=Pakistan). The city of Faisalabad is one of the largest cities in Pakistan which is known for its industrial activities and dense traffic. Therefore, the city has major air pollution problems (PM_2.5_, PM_10_, TSP, CO, NO_2_, SO_2_, etc.). There are a number of recent and comprehensive studies of particulate matter sources, distribution, and chemical composition in Pakistan (Hamid et al., 2023) and in Faisalabad (Shahid et al., 2012; Niaz et al., 2012; Javed et al., 2015, 2016; Bashir et al., 2023). These studies indicate that in Faisalabad brick kilns (Hamid et al., 2023), industry and vehicular emissions, including tire-derived particles (Adachi & Tainosho, 2004), are important sources. The recent work of Bashir et al. (2023) shows that for ten sites in Faisalabad Cd and Pb are the most common heavy metals present in particulate matter, followed by copper, nickel, and then zinc. The daily carbon and sulfur contents of air have been studied for the first time in Pakistan in this work. The aim of this study was to measure pollutant concentrations along the roadsides of the Faisalabad metropolitan area, to calculate seasonal variations, and to determine the potential impact of roadside pollution exposure on human health. We also calculated a novel AQI based on the measurement of six air pollutants defined by the US-EPA (U.S. EPA, 2023), World Health Organization (WHO, 2021), and Pakistan Environmental Protection Agency (Pak-EPA, 2010).

## Materials and methods

### Description of sampling sites

**Fig. 1:**
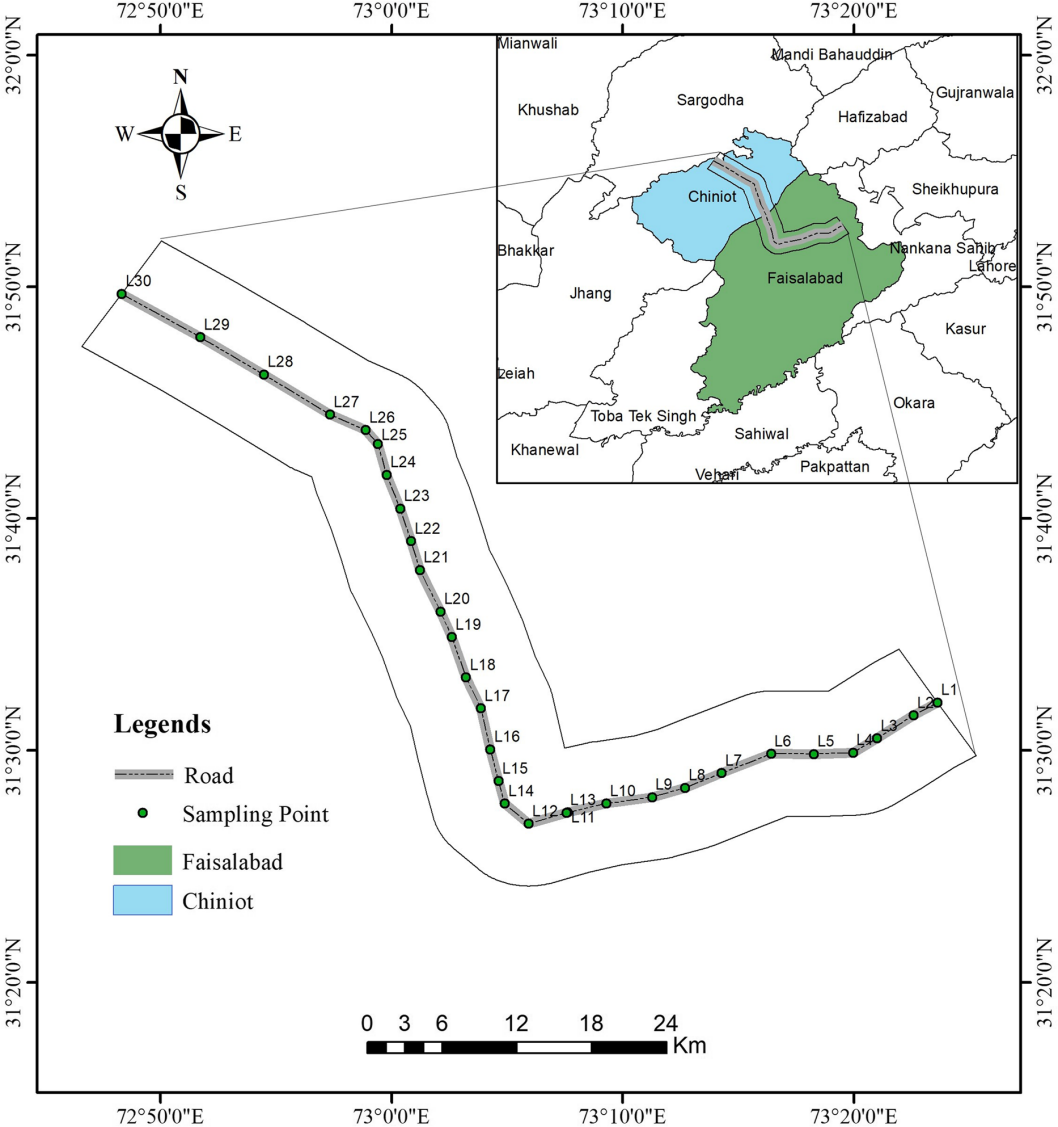
GIS-based map of the study area and sampling points along the selected road

Faisalabad is the third largest city in Pakistan and is a major industrial hub. Thus, the city is popularly known as the Manchester of Pakistan. It is sandwiched between longitudes 73° to 74° east and latitudes 30° to 31.15° north. It has an area of 1230 km^2^. Faisalabad lies 184 m above sea level (Pasha et al., 2015). Figure 1 shows the major districts of the Punjab Province. Our study area lies between district Faisalabad (indicated as green) and district Chiniot (indicated as blue). Based on the preliminary survey, the selected sites have diverse area usage and are representative of urban, dense traffic areas, motorways, industrial areas, railway crossings, bridges, the river, and rural and agricultural areas. The study zone starts from the Sitara Chemical Industries, Sheikhupura Road, Faisalabad (FSD) to Masood Textile Mill, Sargodha Road, FSD via Khurrianwala, Gatwala Forest Park and Nishat Abad Towns and from Masood Textile Mill to Bhianwala, Sargodha Road, Tehsil Lalian to District Chiniot, via Chiniot and Chenab Nagar. The monitoring points (L1, L2, and so on) along the selected roads are shown in Fig. 1.

### Measurement of particulate and gaseous pollution

Detailed description of sampling locations is given in Table 1. Particulate and gaseous pollutants were measured at each location and a 3-km grid distance was maintained between each location. At each location and for each pollutant four concentrations were recorded each hour and these data were used to calculate average hourly concentrations. Micro-Dust Pro Real Time Aerosol Monitors (model HB3275-07, Casella CEL, UK) were used for PM_2.5_, PM_10_, and TSP measurements at each site, with instruments run for 6-h, periods between filter analysis. The instrument could detect 0.001 to 2500 mg m^−3^ at 0.001 mg m^−3^ resolution by absorption of infrared light using angles 12 to 20°. For size of particulate pollution, polyurethane foam filter (PUFF) having a flow rate of 3.5 L/min was used (Javed et al., 2014, 2015). Gaseous pollutants (CO, NO_2_, and SO_2_) were measured using the MIRAN SapphIRe Portable Infrared gas analyzer Model 205B. This instrument detects any compound with absorbance in the wavelength region from 7.7. to 14.1 µm or any of the available fixed band pass filters, such as CO, NO_2_, and SO_2_, hydrocarbons. To operate analyzers and ensure quality control, manufacturer’s Basic User Guidelines or Manuals were followed (https://www.equipcoservices.com/pdf/manuals/thermo_miran_sapphire.pdf).

**Table 1:** Description of monitoring sites, latitude, longitude, and category

Location	Name of the site	Latitude (N)	Longitude (E)	Category
L1	Sitara Chemical Industries, Sheikhupura Road, FSD	31.534637	73.393401	Industrial, Rural
L2	Johal Adda, Sheikhupura Road, FSD	31.525431	73.376036	Vehicular, Rural
L3	Jamia Masjid, Sheikhupura Road, FSD	31.508932	73.349835	Agricultural, Rural
L4	JK Spinning Mill LTD, Sheikhupura Road, FSD	31.498615	73.332378	Industrial, Urban
L5	Hilton Enterprises, Sheikhupura Road, FSD	31.497611	73.304312	Industrial, Urban
L6	Khurrianwala, Sheikhupura Road, FSD	31.497674	73.273322	Vehicular, Urban
L7	Attock Electronic Pump, Sheikhupura Road, FSD	31.483809	73.237662	Vehicular, Urban
L8	Gatwala Forest Colony, Sheikhupura Road, FSD	31.473274	73.211426	Forest, Urban
L9	The Marquee by Dynasty, Sheikhupura Road, FSD	31.466572	73.187611	Commercial Urban
L10	Al Saudia Cash and Carry, Sheikhupura Road, FSD	31.461887	73.154541	Commercial Urban
L11	Sugar Mor, Sheikhupura Road, FSD	31.455601	73.126731	Vehicular
L12	NADRA Office, Sheikhupura Road, FSD	31.447561	73.098668	Vehicular, Urban
L13	Masood Textile, Sargodha Road, FSD	31.455411	73.126052	Industrial, Urban, Vehicular
L14	Rathor Specialist Hospital, Sargodha Road, FSD	31.462003	73.081431	Vehicular, Urban
L15	Bawa Chak Saim Nala, Sargodha Road, FSD	31.478071	73.076935	Vehicular, Urban
L16	Nalka Kohala Stop, Sargodha Road, FSD	31.500977	73.071156	Vehicular, Urban
L17	CITI Housing, Sargodha Road, FSD	31.530443	73.064331	Agricultural, Urban, Vehicular
L18	Shell Pump, 2 chak, Sargodha Road, FSD	31.552809	73.053631	Agricultural, Rural, Vehicular
L19	Police Station, 1 Chak, Sargodha Road, CHT	31.581752	73.043409	Agricultural, Rural, Vehicular
L20	FAST university, Sargodha Road, CHT	31.600165	73.035247	Agricultural, Rural, Vehicular
L21	Jhok Mor, Sargodha Road, CHT	31.629801	73.020458	Agricultural, Vehicular
L22	Patrol Pump Rajoa, Sargodha Road, CHT	31.650542	73.014101	Agricultural, Industrial
L23	Jamia Islamia Imdadia, Sargodha Road, CHT	31.674057	73.006147	Vehicular, Urban
L24	Rice Mill Chiniot, Sargodha Road, CHT	31.698279	72.996413	Industrial, Vehicular, Urban
L25	Qalma Chock, Sargodha Road, CHT	31.720612	72.990144	Vehicular, Urban
L26	Atlas Honda Dealership, Sargodha Road, CHT	31.730522	72.981221	Vehicular, Urban
L27	Kot Meer Shah, Sargodha Road, CHT	31.731661	72.805562	Agricultural, Rural vehicular
L28	Ambassy Mor Chenab Nagar	31.760521	72.917911	Vehicular
L29	Ahmed Nagar, Sargodha Road, Chiniot	31.797343	72.862101	Agricultural, Vehicular
L30	Bhianwala, Sargodha Road, Lalian	31.828532	72.805611	Agricultural Rural Vehicular

### Air quality index (AQI)

AQI is calculated and presented worldwide in order to provide single numbers which describe air quality based on measurements of a number of pollutants. There are a number of ways to calculate AQI (Kumari & Jain, 2017), and we adopted a relatively simple approach based on the PM_2.5_, PM_10_, TSP, CO, NO_2_, and SO_2_ monitoring data available to us. We used the formula described below in accordance with the established standards.

$$\text{Air Quality Index }(\text{AQI})=\frac{1}{4}\times [\frac{{C}_{PM2.5}}{{S}_{PM2.5}}+\frac{{C}_{PM10}}{{S}_{PM10}}+\frac{{C}_{TSP}}{{S}_{TSP}}+\frac{{C}_{CO}}{{S}_{CO}}+\frac{{C}_{NO2}}{{S}_{NO2}}+\frac{{C}_{SO2}}{{S}_{SO2}})\times 100$$

where *C* and *S* showed the concentration of respective pollutant and their standards set by the Pakistan Environmental Protection Agency (Pak-EPA, 2010 and Mir et al., 2024). Further AQI is categorized into 8 categories which are as follows: (0–50) good, (51–100) moderate, (101–150) unhealthy for sensitive groups, (151–200) poor and unhealthy, (201–300) very poor and unhealthy, (301–400) hazardous, (401–500) very hazardous and (> 500) very critical (Pak-EPA, 2010).

### Dust-bound carbon and sulfur contents

Total suspended particulate (TSP) mass samples were collected at each site on micro glass fiber filter papers (47 mm) using a high-volume air sampler (model CF-1001BRL, Hi-Q, USA). These filters were used because they are inert, highly pure, capable of retaining particles down to 0.01 µm size, have high PM collection efficiency (97%), and very low moisture absorption capacity (Method IO-3.1, 1999). Moreover, the protective covering of a Hi-Vol sampler protects the instrument as well as the high-speed motor which acts as a blower. The samples were taken 10 m away from the road at a height of 5 m above ground level. TSP mass was determined gravimetrically from the filters after conditioning in desiccator for 24 h at 45 ± 5% relative humidity and 23 ± 3 °C temperature. After the entire process, each filter paper containing PM was stored in an aluminum foil and sent to the lab for carbon and sulfur analysis (US EPA, 2015). One gram dust was used to analyze carbon and sulfur contents using Infrared Carbon Sulfur Analyzer CS996 at the Punjab Bioenergy Institute, Faisalabad (SPM, 1999).

### Human blood sampling

Human blood samples from 20 individuals (numbered P1 to P20) from the selected sampling sites were obtained once in each of the four seasons. Contact phone numbers were obtained to ensure that the same individuals could be contacted and resampled. Fresh venous blood samples were collected using sterile syringes Luer-lok tip with BD Precision Glide Needle 23G × 1W (0.6 mm × 25 mm) and stored in 5 ml K2 EDTA (K2E) 5.4 mg blood collection tubes at 4 °C till further analysis. A questionnaire-based survey was conducted to gain information of the sampled individuals to obtain their demographic status, i.e., food intake, drinking water source, and major air pollutant exposure (proximity to known sources). Medical information of the targeted population was also collected.

### Digestion of blood samples for analysis of Cd and Pb on flame atomic absorption spectrophotometer

The blood samples were digested and analyzed using conventional methods (see Mahmood et al., 2014). For this 0.5 mL of a whole blood sample was taken in a Pyrex flask. The digestion mixture (nitric acid and hydrogen peroxide) was prepared using the 2:1 ratio, by volume. Then 3 mL of digestion mixture was added to the flask containing the blood sample and allowed to stand for 10 min. Flasks were then heated on a hot plate at 60–70 °C for 1 to 2 h. After that 2 mL of nitric acid was added with a few drops of hydrogen peroxide at 80 °C until a clear digest is obtained. The excess acid was allowed to evaporate and then diluted with 0.1 *N* nitric acid. Then these samples were transferred to a 25-mL volumetric flask and double distilled water was added to make up the volume. The blank sample was also prepared (Popoola et al., 2019) using the same procedure without the blood sample. Cd and Pb were analyzed on atomic absorption spectrophotometer (AAS). The coordinates of each sampling location are already presented against the location number in Table 1. Details of each patient are given in Table 2 along with their occupation.

**Table 2:** Details of individuals selected for blood sampling for heavy metal analysis

Sr. No	Person ID	Location	Occupation
1	P1	L1	Open charcoal cooking stall holder
2	P2	L3	General store owner/worker
3	P3	L5	General store owner/worker
4	P4	L7	Open charcoal cooking stall holder
5	P5	L8	General store owner/worker
6	P6	L9	General store owner/worker
7	P7	L11	Barber
8	P8	L13	General store owner/worker
9	P9	L14	Open charcoal cooking stall holder
10	P10	L15	General store owner/worker
11	P11	L17	Barber
12	P12	L18	General store owner/worker
13	P13	L20	Open charcoal cooking stall holder
14	P14	L23	Barber
15	P15	L24	General store owner/worker
16	P16	L25	Auto mechanic
17	P17	L26	Open charcoal cooking stall holder
18	P18	L28	Auto mechanic
19	P19	L29	Barber
20	P20	L30	General store owner/worker

### GIS mapping

Inverse distance weighted (IDW) method was used using ARC GIS 10.8 for interpolation of the concentration of air pollutants at each site. In IDW, technique sampling points were joined together and weighted according to their distance from the point being interpolated, hence creating a surface grid (Ahmad et al., 2012; Briggs et al., 1997; Parveen, 2012).

### Statistical analysis

Relationship between measured air pollutants were explored using correlation analysis. Descriptive statistics were applied to the data set and mean, standard deviation (St. Dev.), Coefficient of variation, skewness, kurtosis, minimum, and maximum were calculated for each pollutant. Excel 365, JASP 0.8.5.1, Origin Pro, and SPSS software were used for data processing and visualization. Skewness and kurtosis values indicated normal distributions. These values are shown in the Supplementary Information.

## Results and discussions

### Seasonal and spatial variation of PM_2.5_, PM_10_, TSP, and CO

**Fig. 2:**
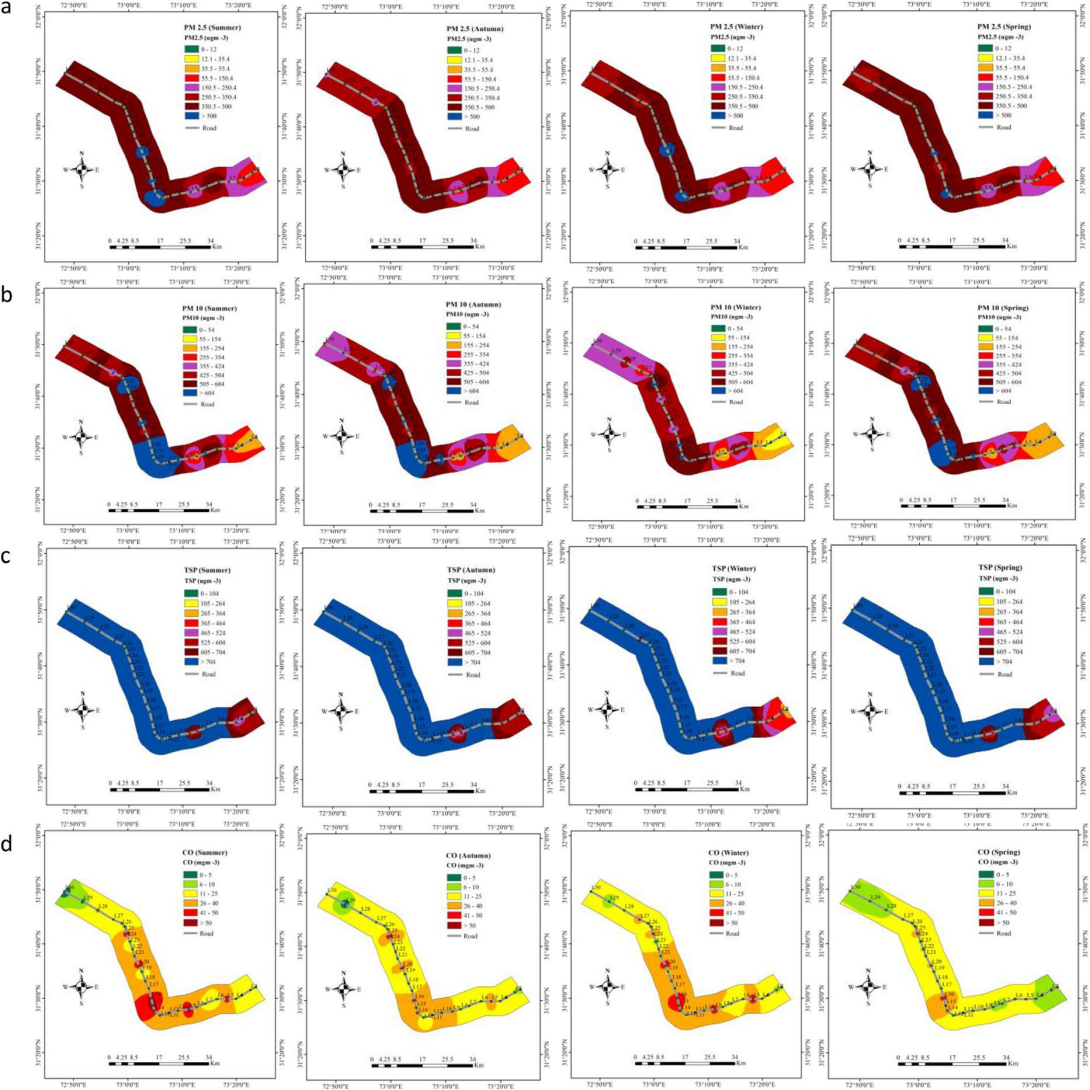
Interpolation of seasonal variations of **a** PM_2.5_, **b** PM_10_, **c** TSP, and **d** CO concentrations in Summer 2019, Autumn 2019, Winter 2020, and Spring 2020 (left to right)

Each particulate pollutant concentration was categorized into seven colors which represent the quality of air, i.e., green (good), yellow (marginal), orange (unhealthy for sensitive groups), red (poor or unhealthy), purple (very poor or very unhealthy), maroon (hazardous), dark maroon (very hazardous) and blue (very critical). The concentration ranges which equate to each color are shown in Fig. 2 (Gurjar et al., 2008; U.S. EPA, 2012). The lighter colors represent the lowest concentration and darker colors represent the highest concentrations (Ahmad et al., 2012). These categories were used to create a seasonal and spatial distribution of PM_2.5_, PM_10_, and TSP.

The spatial interpolation maps during summer, autumn, winter, and spring, are shown in Fig. 2. This Figure clearly indicates the spatial variability of PM_2.5_ concentrations along the sampling roads; these ranged across five concentration categories from poor–unhealthy (55.5–150.4 μg m^−3^) to very critical (> 500 μg m^−3^). During summer lower concentration was found at locations L1 to L4—shown as red color in Fig. 2. Higher concentrations were found at locations L12, L14, L16, L19, and L20—shown in blue color. The blue color showed that the concentration of PM_2.5_ was above 500 μg m^−3^ which is very critical. Seasonal distribution shows that concentrations of PM_2.5_ in each season were above 55.5 μg m^−3^ which is categorized as poor and unhealthy. TSP showed similar spatial patterns as for PM_2.5_ (lower values at locations 1 to 4 and higher values at the other locations). As would be expected TSP concentrations were higher than those for PM_2.5_ and PM_10_ and their magnitudes were very critical at most locations in all four seasons (Fig. 2). The PM_10_ concentrations showed slightly different spatial patterns with some of the locations in the middle of the transect showing very critical (blue) values in summer, spring and autumn. The CO concentrations showed a similar spatial pattern to that seen for PM_10_ (Fig. 2). These maps clearly visualize the area of low, moderate, and higher concentration of PM_2.5_, PM_10_, TSP, and CO during each season. Poor road structures, poor maintenance of vehicles, and congested road with high traffic density are the reasons of higher concentrations of pollutants along the selected road (Ashraf et al., 2010; Li et al., 2023; Piracha & Chaudhary, 2022; Xu et al., 2022). These results, with very few periods of air quality at moderate or good levels, suggest that better management of air quality in the mapped areas is important for stakeholders.

For NO_2_ our observed seasonal average concentrations were Summer 2019–71.98 µg m^−3^, Autumn 2019–53.02 µg m^−3^, Winter 2020–57.04 µg m^−3^, and Spring 2020–35.02 µg m^−3^ (see Supplementary Table 1). Thus, all seasonal average NO_2_ concentrations exceed the 2021 WHO Air Quality Guideline level which is an annual average of 10 µg m^−3^. With values at 64.7, 42.07, 50.82 and 39.28 µg m^−3^ for Summer 2019, Autumn 2019, Winter 2020, and Spring 2020, respectively (see Supplementary Table 1), our measured seasonal SO_2_ concentrations also exceeded, or in the case of spring, were very close to the WHO AQG level of 40 µg m^−3^.

### Seasonal pattern of AQI based on six studied pollutants at each location

**Fig. 3:**
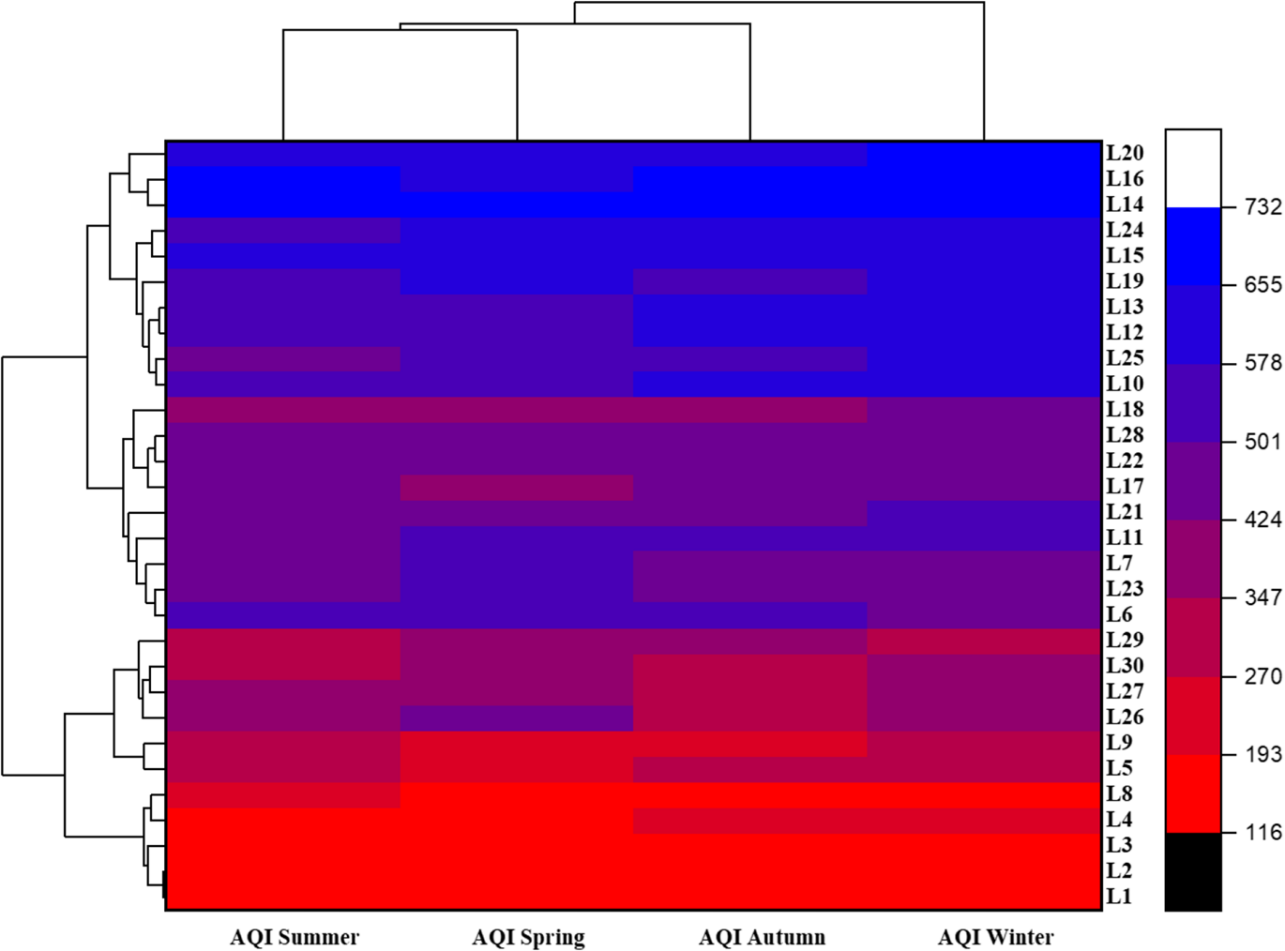
Heat map with dendrogram of seasonal variation in AQI based on PM_2.5_, PM_10_, TSP, CO, NO_2_, and SO_2_

Figure 3 shows the seasonal variation of the air quality index (AQI) at each location based on PM and gaseous pollutant concentrations. The red color indicates a lower AQI (116), while blue indicates a higher AQI (732) during different seasons at each location. The overall air quality was categorized as very poor and unhealthy for location 2 during each season to very critical for L16, L14, etc. as shown in Fig. 3. AQI of summer and spring were clustered in the same group associated with autumn and winter AQI levels. The primary clusters were found between L1 and L2, L4 and L8, L5 and L9, L27 and L30, L22 and L28, L10 and L25, L12 and L13, L15 and L24, L14 and L16 which shows that these locations have the same anthropogenic source of emission, i.e., vehicles.

### Summary of means and standard errors of particulate and gaseous pollutants

**Fig. 4:**
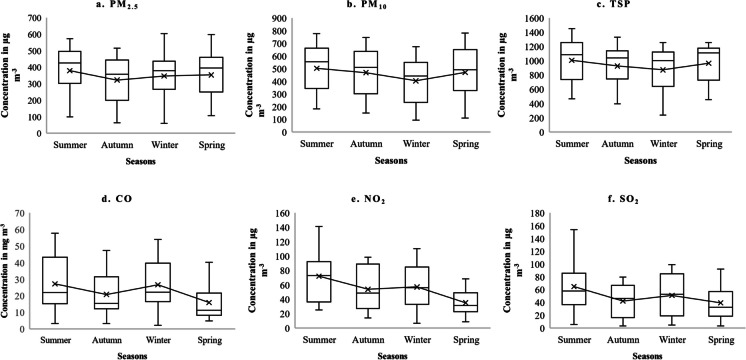
Seasonal variation of all pollutants with means (*X*) and median, interquartile range, maximum and minimum values in box notation

The three pollutants (PM_2.5_, PM_10_, TSP) all showed the highest concentrations in summer, and were lowest in autumn or winter (see Fig. 4). The seasonal fall in PM concentrations (PM_2.5_, PM_10_, and TSP) and gaseous pollutant (CO, NO_2_, and SO_2_) from summer to autumn could reflect “washout” associated with monsoon rainfall which occurs from July through to September and the winter rise in gaseous pollutants probably arise from increased fuel use. But we feel that the high PM concentrations recorded here throughout the year reflect that the roads are unmade-up, in poor condition and have bare ground along the roadside. The high PM and gaseous pollutant values which we recorded result from the high and whole-year emissions from Faisalabad’s roads, local chemical and textile industries and the wood-based power plant. CO, SO_2_, and NO_2_ also all had the highest concentrations in summer or spring. NO_2_, SO_2_ and CO had the lowest concentrations in winter (see Fig. 4). Standard deviation (St. Dev.), coefficient of variance (CV), skewness, kurtosis, minimum, and maximum of particulate and gaseous pollutants are given in (Supplementary Table 1).

### Seasonal variation of carbon and sulfur contents bound with road dust

**Fig. 5:**
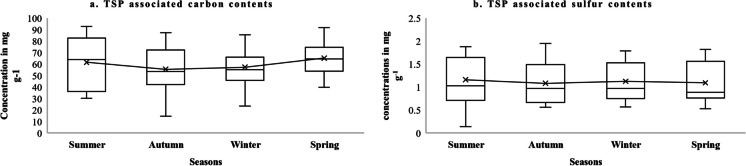
The seasonal values of carbon (**a**) and sulfur (**b**) content of dust samples in mg g^−1^. Means (*X*) and median, interquartile range, maximum and minimum values in box notation

Dust-bound carbon content (CC) was highest in the summer and spring (Fig. 5a). The lowest values of CC were at locations L8 and L9 (14.67 ± 0.94 and 23.00 ± 1.63 mg g − ^1^ respectively (means and SE) which are forest and a commercial urban area where the road was in good condition (see Table 1). The highest CC was 87.33 ± 2.49 and 91.67 ± 1.25 mg g^−1^ at locations L16 and L17, respectively. Both these locations have vehicular emissions in an urban setting where agricultural practices are also involved. Here the road structure is also poor where vehicles use more fuel and produce more exhaust along with unburnt particles. When vehicles pass the road, dust is re-suspended and increases the pollution load in the surroundings. In a Chinese study, CC was analyzed in street dust, and it was found that CC ranges from 5 to 71 mg g^−1^ (Bandowe et al., 2019). CC was mainly generated from the exhaust of vehicles and coal and wood burning in industries and crop burning (Lin et al., 2020). The researchers found that CC concentration was higher near the road trunks (Ma et al., 2019) and near the sources of fossil fuel burning (Han et al., 2009). It was also found that where CC was high there was also higher heavy metal contamination (Pan et al., 2017).

Seasonal variations of sulfur content (SC) are given in Fig. 5b. There was little seasonal variation in SC but values were lower at locations L30, L26, L5, and L5 where hard (carpeted) road was present (SC values of 0.14 ± 0.024, 0.56 ± 0.016, 0.57 ± 0.012, and 0.53 ± 0.022, mg g^−1^, respectively). L26 and L30 are in the city of Chiniot (see Table 1) and heavy traffic (trucks, loaders, etc.) is diverted through a bypass present near the Iqbal Rice Mill to Lahore Road and Jhang road. Due to this tire abrasions as well as re-suspension of dust were reduced. SC were highest at locations L20, L16, L10, and L20 (1.88 ± 0.076, 1.95 ± 0.009, 1.31 ± 0.109, and 1.82 ± 0.021 mg g^−1^ respectively). These locations have urban vehicular emissions and commercial and agricultural activities (see Table 1). This variation is thought to be caused by the type of vehicle passing at the time of sampling. If a truck is passing at the time of sampling and its exhaust was intensely black, then high SC values were recorded. This suggests that SC content of roadside dust depends upon the type and condition of vehicle engine. The sulfur content of roadside dust depends upon the amount of sulfur present in fuel (Saiyasitpanich et al., 2005). The sulfur content of gasoline and diesel varies considerably (c 0.3 to 1% by weight) and work in Pakistan has shown that for diesel S concentrations can be as much as 2.5 times higher than shown on supplier certification (Hadis et al., 2011). It is the need of the hour to reduce SC from fossil fuels which would significantly protect the urban environments particularly where traffic density is high (Saleh, 2020).

### Human health survey in Faisalabad Metropolitan

**Fig. 6:**
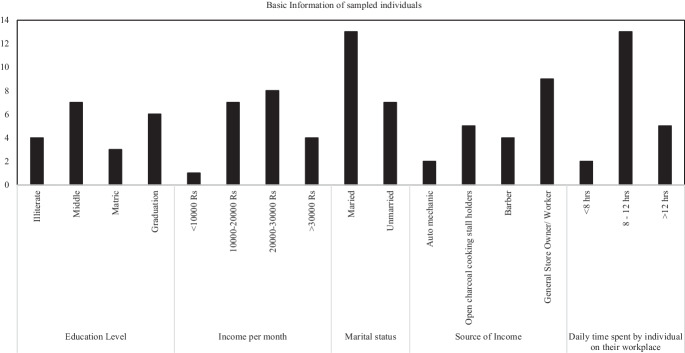
Basic information collected from each sampled individual (from left to right -educational level, income per month, marital status, source of income, and daily time spent in the workplace)

Twenty males were selected for blood sampling due to the unavailability of females by the roadsides. Figure 6 shows the information collected from each individual from whom blood samples were collected. Informed consent was obtained from those individuals. Of the persons sampled, 4 were illiterate, 7 had passed 8th standard, 3 had passed 10th standard and 6 were graduates (see Fig. 6).

Four income levels were marked (1 ≤ Rs. 10,000; 2 = Rs. 10,000–20,000; 3 = Rs. 20,000–30,000; and 4 ≥ Rs. 30,000). Only one person had an income of less than Rs. 10,000/month. Seven had incomes in the range of Rs. 10,000–20,000/month, eight in the range of Rs. 20,000–30,000/month and four were above Rs. 30000/month. Sixty-five percent of the individuals studied were married. 10% were bike mechanics, 25% were open charcoal cooking stall holders, 20% were barbers and 45% ran general stores or small shops like (tea shop, grocery outlet, welding shop, etc.). Ten percent of the studied population spent less than 8 h, 65% of the studied population spent 8–12 h and only 25% spent more than 12 h at their workplace by the roadside. The full questionnaire used is shown in the Supplementary Information (Table 2). Participant responses were recorded using the scale: 1, very low; 2, low; 3, medium; 4, high; and 5, very high (see Supplementary).

### Concentration of Cd and Pb in human blood

**Fig. 7:**
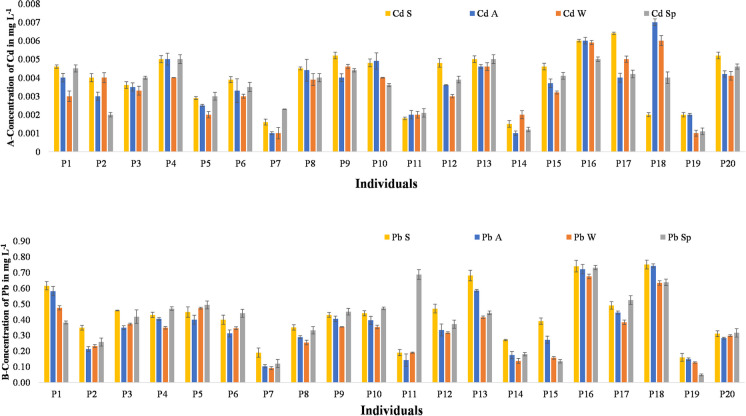
**A** Concentration of Cd and **B** concentration of Pb. Data show the mean blood concentrations and standard errors for 20 individuals (P1 to P20) sampled in Summer, Autumn 2019, Winter, and Spring 2020 (S, A, W, and Sp)

The concentration of Cd and Pb in mg L^−1^ in the blood of sampled individual during each season is given in Fig. 7A and B. Each digested blood sample was analyzed 3 times to get the means and standard deviations. The lowest concentrations of Cd in all seasons were 0.001 mg L^−1^ shown in individuals P7, P14, and P19, and the individual with the next lowest blood Cd values was individual P11 (c 0.002 mg L^−1^). These individuals (P7, P11, P14, and P19) all work in barber’s shops (see Table 2) which have closed glass doors, thereby having lower exposure to road emissions. The highest blood concentrations of Cd in summer, autumn, winter, and spring were 0.006, 0.007, 0.006, and 0.005 mg L^−1^ in individuals P17, P18, P18, and P4, respectively. P4 and P17 had BBQ shops while P18 had an auto mechanic shop (see Table 2). The individuals with the highest Cd contents were from L11, L13, L16, L18, and L28 (see Table 2) and the air quality of these locations was very critical with dark blue color as shown in Fig. 3. These individuals all had open shops and were directly exposed to bad air.

The lowest individual blood concentrations of Pb in summer, autumn, winter, and spring were 0.16, 0.1, 0.09, and 0.05 mg L^−1^ and were measured in individuals P19, P7, P7, and P19, respectively. As with Cd, the individuals who work in barber shops with glass doors had lower roadside exposure (P7 and P19) were among this group. The highest blood concentrations of Pb in summer, autumn, winter, and spring were 0.75, 0.74, 0.67, and 0.73 mg L^−1^ in individuals P18, P18, P16, and P16, respectively (Fig. 7B). P16 and P18 were both auto mechanic and showed the highest concentration of Pb in their blood. They were not only exposed to very critical level of air pollution at L25 and L28 (see Fig. 3) but also worked with petrol and diesel with bare hands. The higher Pb concentrations in the blood of these individuals were probably the result of surface absorption of Pb into the bloodstream from vehicle paints as Pb was banned in gasoline at the beginning of this century. It has been shown that roadside vehicles harm humans in many ways. The major route of entry is from air through the nose and adults and children are both at risk of adverse health effects (Ahmad et al., 2019) due to indoor (Tham, 2016) and outdoor (Leung, 2015) air pollution.

Twenty is a small sample size. Moreover, participants were not randomly selected but on the basis of proximity to our sampling locations. A further limitation is that we had no history of exposure for the sampled individuals which may have included other sources of Cd and Pb; for example, smoking or maternal exposure which is known to be important in human Cd and Pb impacts (Wang et al., 2019). However, the results clearly revealed blood Cd and Pb concentrations which are above the permissible limit set by (WHO, 1996). The permissible limit for Cd content in blood is 0.001 mg L^−1^and for Pb it is 0.1 mg L^−1^ (Alli, 2015). Cd creates reactive oxygen species in the blood due to which oxidative stress increases and body defensive systems are disturbed. Its toxicity increases with the increase of age of human. As there is no chelating agent present to convert Cd into less toxic form it is not excreted by the body and accumulates over time. Further, this may lead to DNA damage and the occurrence of cancer in humans. As the primary source of Cd is through inhalation and ingestion, vehicles enhance the chances of Cd accumulation through oral activity (Alengebawy et al., 2021; Suhani et al., 2021). Pb is also very toxic. Lead is directly involved with the blood hemoglobin. It inhibits the enzyme δ-aminolaevulinic acid dehydratase (ALAD), coproporphyrinogen, and ferro chelatase and reduces the activity of red blood cell. Therefore, excess Pb damages the blood vessels affecting the oxygen supply to the whole body. It causes mitochondrial degeneration in the kidney cells and hence disturbs the function of the kidney. Slow and continuous exposure of Pb can cause liver toxicity in humans (Lopes et al., 2021; Yan et al., 2022). Thus, Cd and Pb originating from roadside vehicles cause damage to human health.

## Conclusions

The data presented here show that particulate pollutant concentrations along the roadsides of Faisalabad are above the WHO guideline. Moreover, as they contain Cd and Pb (Shahid et al., 2012; Niaz et al., 2012; Javed et al., 2015, 2016; Bashir et al., 2023), they have the potential to be harmful to human health. This data presents for the first time confirmation of the levels of exposure in Faisalabad. The concentrations of NO_2_ were found to be higher than the WHO AQG level of 10 µg m^−3^ at locations L9, L10, L13, L14, L15, L16, L20, L21, and L24. SO_2_ concentrations measured as seasonal averages also exceeded or were very close to (spring) the WHO, 2021 AQG annual average value of 40 µg m^−3^. The blood Pb and Cd analysis presented here suggests that levels in the air at our study locations are high and that people are being exposed to unsafe levels. Given the small sample number and other sampling limitations (see above) the blood Pb and Cd concentrations, although not showing a statistical association, are a cause for concern in that they indicate the potential for health impacts. The concentrations of air pollutants are increasing day by day and can be damaging to human health. Our mapping work indicates that exhaust from vehicles and industry along the road is causing an increase in gaseous pollution. Emissions are reducing air quality along the roadside and potentially posing a threat to human health, and probably also causing deleterious effects on the ecological system.

### Supplementary Information

Below is the link to the electronic supplementary material.

Supplementary file1 (DOCX 345 KB)

## Data Availability

All data is presented in the manuscript.
