# Supplementary material for: Particulate and gaseous air pollutants exceed WHO guideline values and have the potential to damage human health in Faisalabad, Metropolitan, Pakistan
Source: Environ Monit Assess. 2024 Jun 25;196(7):659. doi: 10.1007/s10661-024-12763-3 (PMC11199306; doi:10.1007/s10661-024-12763-3)
Supplement: Supplementary file 1 — Supplementary file1 (DOCX 345 KB) [file 10661_2024_12763_MOESM1_ESM.docx]

Supplementary Information

**Supplementary Table 1** Descriptive statistics of PM_2.5_, PM_10_, TSP, CO, NO_2_ and SO_2_ in Summer, Autumn, Winter, and Spring along the sample transect of Faisalabad’s roads.

| **Seasons** | **Pollutants** | **Units** | **Mean** | **St. Dev** | **CV**  **prop** | **Skewness** | **Kurtosis** | **Min** | **Max** |
| --- | --- | --- | --- | --- | --- | --- | --- | --- | --- |
| Summer | **PM_2.5_** | µg m^-3^ | 378.13 | 149.51 | 0.4 | -0.7 | -0.79 | 98 | 572 |
|  | **PM_10_** | µg m^-3^ | 502.70 | 187.16 | 0.37 | -0.35 | -1.23 | 183 | 776 |
|  | **TSP** | µg m^-3^ | 1005.73 | 310.21 | 0.31 | -0.52 | -1.01 | 465 | 1453 |
|  | **CO** | mg m^-3^ | 27.18 | 17.72 | 0.65 | 0.49 | -1.06 | 3.25 | 57.68 |
|  | **NO_2_** | µg m^-3^ | 71.98 | 36.56 | 0.51 | 0.39 | -0.94 | 25.22 | 141.21 |
|  | **SO_2_** | µg m^-3^ | 64.70 | 43.00 | 0.66 | 0.95 | 0.75 | 5.62 | 168.21 |
| Autumn | **PM_2.5_** | µg m^-3^ | 321.33 | 144.62 | 0.45 | -0.57 | -1.04 | 63 | 514 |
|  | **PM_10_** | µg m^-3^ | 468.63 | 193.05 | 0.41 | -0.26 | -1.22 | 149 | 747 |
|  | **TSP** | µg m^-3^ | 926.03 | 273.36 | 0.3 | -0.65 | -0.84 | 396 | 1330 |
|  | **CO** | mg m^-3^ | 21.66 | 13.66 | 0.63 | 0.87 | -0.51 | 3.23 | 52.57 |
|  | **NO_2_** | µg m^-3^ | 53.65 | 29.9 | 0.56 | 0.27 | -1.48 | 13.87 | 98.21 |
|  | **SO_2_** | µg m^-3^ | 42.07 | 26.33 | 0.63 | -0.11 | -1.53 | 3.13 | 79.36 |
| Winter | **PM_2.5_** | µg m^-3^ | 345.93 | 152.69 | 0.44 | 2-0.48 | -0.59 | 59 | 602 |
|  | **PM_10_** | µg m^-3^ | 403.3 | 185.07 | 0.46 | -0.32 | -1.13 | 93 | 674 |
|  | **TSP** | µg m^-3^ | 871.3 | 311.14 | 0.36 | -0.68 | -0.91 | 234 | 1256 |
|  | **CO** | mg m^-3^ | 26.64 | 14.75 | 0.55 | 0.38 | -0.9 | 2.21 | 54.04 |
|  | **NO_2_** | µg m^-3^ | 57.04 | 31.92 | 0.56 | 0.03 | -1.23 | 6.57 | 110.21 |
|  | **SO_2_** | µg m^-3^ | 50.82 | 33.03 | 0.65 | 0.04 | -1.51 | 4.29 | 99.24 |
| Spring | **PM_2.5_** | µg m^-3^ | 352.2 | 140.64 | 0.4 | -0.55 | -0.76 | 105 | 597 |
|  | **PM_10_** | µg m^-3^ | 469.87 | 186.53 | 0.4 | -0.31 | -0.92 | 110 | 781 |
|  | **TSP** | µg m^-3^ | 964.07 | 276.89 | 0.29 | -0.8 | -1.02 | 454 | 1255 |
|  | **CO** | mg m^-3^ | 15.93 | 11.06 | 0.69 | 1.34 | 1.27 | 4.74 | 47.81 |
|  | **NO_2_** | µg m^-3^ | 35.02 | 17.36 | 0.5 | 0.33 | -0.87 | 8.54 | 68.27 |
|  | **SO_2_** | µg m^-3^ | 39.28 | 26.39 | 0.67 | 0.6 | -0.61 | 3.21 | 92.29 |

**Supplementary Table 2** Questions asked from each individuals

| Survey Questions | Scale | | | | |
| --- | --- | --- | --- | --- | --- |
|  | **1** | **2** | **3** | **4** | **5** |
| Q 1. Major source of air pollution | | | | | |
| 1. Industrial (chemical, textile, oil refinery) Exhaust | 0 | 1 | 2 | 3 | 14 |
| 1. Vehicular Exhaust | 0 | 3 | 0 | 7 | 10 |
| 1. Crop Burning | 0 | 3 | 0 | 7 | 10 |
| 1. Use of Chemicals in Agriculture | 0 | 0 | 1 | 8 | 11 |
| 1. Domestic fossil fuel burning | 4 | 5 | 1 | 0 | 10 |
| 1. Brick kiln | 0 | 0 | 0 | 0 | 20 |
| Q 2. Effect of air pollution | | | | | |
| 1. Difficulty in Breathing | 0 | 0 | 0 | 0 | 20 |
| 1. Respiratory diseases | 10 | 0 | 0 | 0 | 10 |
| 1. Skin Allergy | 1 | 7 | 7 | 0 | 5 |
| 1. Emphysema | 0 | 0 | 0 | 5 | 15 |
| 1. Lung Cancer | 0 | 1 | 10 | 4 | 5 |
| 1. Poor Visibility | 0 | 0 | 6 | 7 | 7 |
| 1. Eye Irritation | 3 | 4 | 2 | 5 | 6 |
| 1. Asthma Indices | 0 | 1 | 9 | 6 | 4 |
| Q 3. Is Air pollution injurious for following natural resources? | | | | | |
| 1. Atmosphere | 0 | 0 | 0 | 7 | 13 |
| 1. Water (River, Lakes, Canals etc) | 0 | 0 | 6 | 12 | 2 |
| 1. Land/Agricultural Soil | 0 | 0 | 9 | 9 | 2 |
| 1. Humans | 0 | 1 | 5 | 7 | 7 |
| Q 4. Suggestions to improve air | | | | | |
| 1. Individual level cleaning practices according to Islam? | 0 | 0 | 0 | 0 | 20 |
| 1. Keep your surrounding clean without waiting for government? | 0 | 0 | 0 | 0 | 20 |
| 1. Should Energy efficient technologies be used? | 0 | 0 | 0 | 0 | 20 |
| 1. Polluter pay principle should be implemented | 4 | 0 | 0 | 0 | 16 |
| 1. Public transport should be used instead of private vehicles? | 0 | 0 | 0 | 0 | 20 |
| 1. Enforcement of air pollution control laws with strict fines? | 0 | 0 | 0 | 0 | 20 |


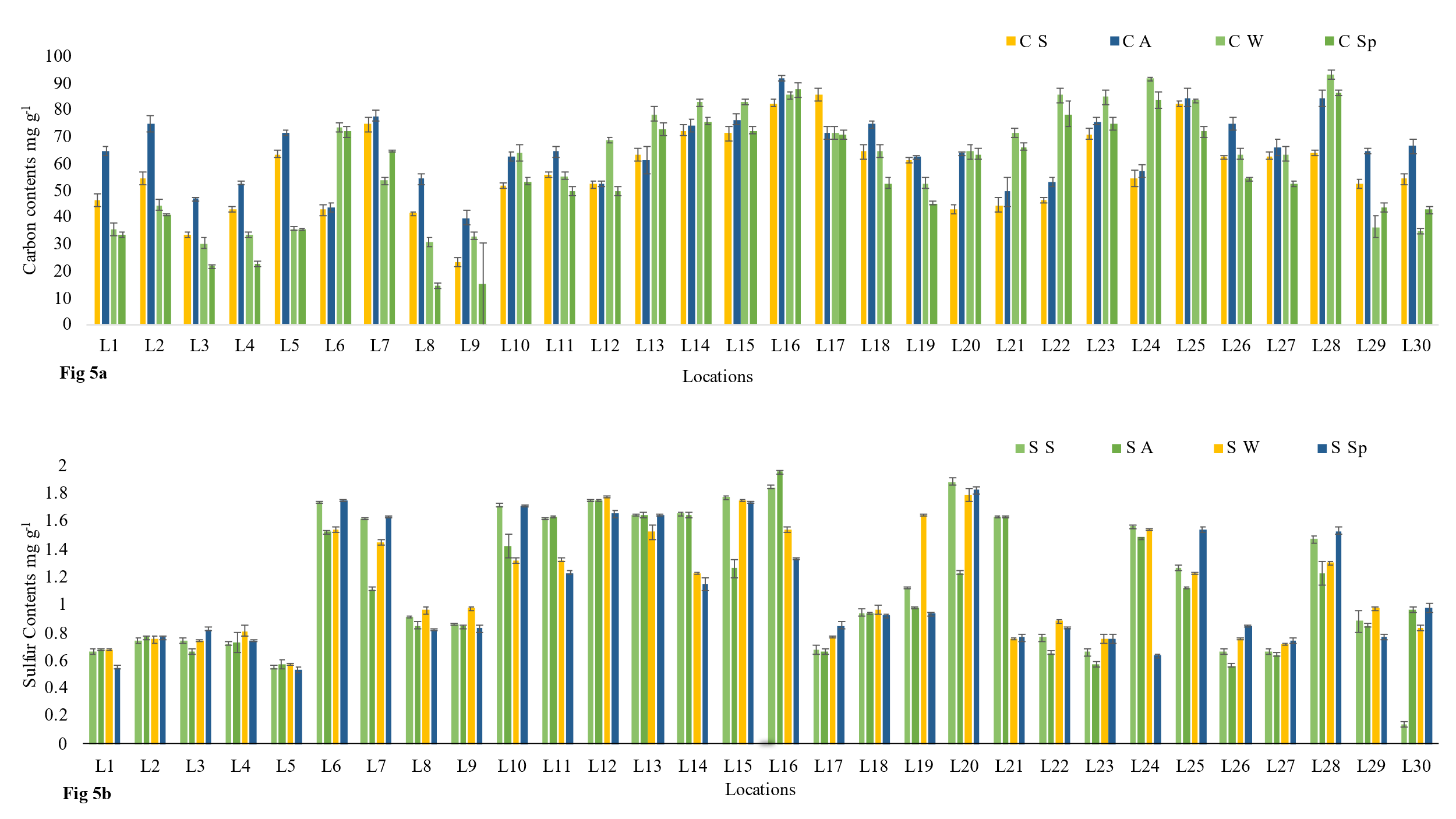


Carbon and Sulfur contents in collected dust samples from each location in mg g^-1^. Individual location data as used for Figs 5a and 5b. Site means and SE are shown CS and SS are Summer values for carbon and sulfur, CA and SA are Autumn values, CW and SW are Winter values and CSp and SSp are Spring values.
